# Growth and Photosynthetic Characteristics of Toxic and Non-Toxic Strains of the Cyanobacteria *Microcystis aeruginosa* and *Anabaena circinalis* in Relation to Light

**DOI:** 10.3390/microorganisms5030045

**Published:** 2017-08-04

**Authors:** M. Ashraful Islam, John Beardall

**Affiliations:** School of Biological Sciences, Monash University, Clayton VIC 3800, Australia; john.beardall@monash.edu

**Keywords:** cyanobacteria, growth, physiology, toxin, energy investment, strain

## Abstract

Cyanobacteria are major bloom-forming organisms in freshwater ecosystems and many strains are known to produce toxins. Toxin production requires an investment in energy and resources. As light is one of the most important factors for cyanobacterial growth, any changes in light climate might affect cyanobacterial toxin production as well as their growth and physiology. To evaluate the effects of light on the growth and physiological parameters of both toxic and non-toxic strains of *Microcystis aeruginosa* and *Anabaena circinalis*, cultures were grown at a range of light intensities (10, 25, 50, 100, 150 and 200 µmol m^−2^ s^−1^). The study revealed that the toxic strains of both species (CS558 for *M. aeruginosa* and CS537 and CS541 for *A. circinalis*) showed growth (µ) saturation at a higher light intensity compared to the non-toxic strains (CS338 for *M. aeruginosa* and CS534 for *A. circinalis*). Both species showed differences in chlorophyll *a*, carotenoid, allophycocyanin (APC) and phycoerythrin (PE) content between strains. There were also differences in dark respiration (R_d_), light saturated oxygen evolution rates (P_max_) and efficiency of light harvesting (α) between strains. All other physiological parameters showed no statistically significant differences between strains. This study suggest that the different strains respond differently to different light habitats. Thus, changes in light availability may affect bloom intensity of toxic and nontoxic strains of cyanobacteria by changing the dominance and succession patterns.

## 1. Introduction

Cyanobacteria play significant roles in the nitrogen, carbon and oxygen dynamics of many aquatic ecosystems. They are major bloom-forming organisms in freshwater ecosystems all over the world and blooms cost millions of dollars annually in lost income and amenities [1]. According to Connolly et al. [2], the predicted cost of nutrient-induced cyanobacterial blooms in just a single system in Australia, the Gippsland Lakes of Victoria, has been estimated to be $256 million over the next 20 years. Globally, these economic costs are predicted to increase over time, due to a warming climate increasing the incidence of algal blooms [3,4]. A number of factors are responsible for the expansion of harmful cyanobacterial blooms including nutrient enrichment, often associated with agricultural activity, and increased surface water temperature due to global climate change [5,6,7]. Nutrient, especially phosphorus (P), enrichment in freshwaters sometimes causes some cyanobacteria to dominate phytoplankton populations [5,8]. Since cyanobacteria have the ability to fix nitrogen, phosphorus (P) enrichment in freshwater is sometimes a major driver for cyanobacterial dominance [8]. However, many cyanobacteria do not fix nitrogen gas and their bloom formation may be limited by the availability of fixed nitrogen in the water column [9,10]. Several genera of cyanobacteria are responsible for potent toxin production, of which most are bloom forming. The most widespread cyanobacterial toxins are hepatotoxins [11] followed by neurotoxins such as saxitoxin. *Anabaena* is the most prevalent toxic cyanobacterial genus throughout the world, some genera of which produce microcystins (MCYs), anatoxin-a and anatoxin-a(S) or cylindrospermopsin (CYN), while others, mainly strains of *Anabaena circinalis*, produce a saxitoxin (STX). While *Anabaena circinalis* produces saxitoxin, *Microcystis,* another widespread genus, mostly produces microcystin (MCY).

Perhaps the most significant factor controlling algal growth is light. Given that toxin production requires an investment in energy and resources, changes in light climate might affect cyanobacterial toxin production as well as their growth and physiology. Light also plays a role in the transition of toxic to non-toxic strains during blooms [12]. In some cyanobacterial genera, growth-saturating light intensities are also those in which intracellular toxin concentrations are highest [13], whereas for other genera maximum cyanotoxin production occurs at irradiances greater [14,15] or lower [16] than those required for maximum growth. Clearly, the effect of light on toxin production is species-specific. A number of studies [17,18,19,20,21] have been performed on cyanobacterial growth and physiology in terms of light climate, but there have been few investigations on the effects of light conditions on toxin production or indeed the consequences of toxin production to energetics and light use efficiency of cyanobacteria. 

Therefore, in this paper we present the results of investigations of growth and physiological characteristics of both toxic and non-toxic strains of the cyanobacteria *Anabaena circinalis* and *Microcystis aeruginosa* in relation to light environment.

## 2. Materials and Methods

### 2.1. Strains and Culture Conditions

*A. circinalis* and *M. aeruginosa* strains were obtained from the CSIRO Marine and Atmospheric Research, Hobart, Australia (Australian National Algae Culture Collection). Strains CS338 (non-toxic) and CS558 (toxic) were originally isolated from Burrinjuck Dam, New South Wales and Shepparton, Victoria respectively, while strains CS534 (non-toxic), CS537 (toxic) and CS541 (toxic) were isolated, respectively, from Fitzroy River, Rockhampton, Queensland, Mount Bold Reservoir, South Australia and Tullaroop Reservoir, Victoria. Subcultures of the isolates were maintained in 250 mL Erlenmeyer flasks containing 120 mL MLA medium [22] in a controlled environment room (25 °C) under a 12:12 h dark:light cycle with a photon flux of photosynthetically active radiation (PAR) of between 40–50 µmol photons m^−2^ s^−1^. The cell concentration at inoculation was 1 × 10^5^ cell mL^−1^. The flasks were shaken gently several times every day to ensure homogenous exposure of cells to the light environment.

To characterise the response of growth to light conditions, strains were grown at six photon flux levels (10, 25, 50, 100, 150 and 200 µmol photons m^−2^ s^−1^). Light was supplied from cool-white fluorescent tubes (Philips, TLD 36W, Amsterdam, The Netherlands) and different intensities maintained by neutral density filters (shade cloth). Cells were grown in 30 mL Nalgene bottles containing MLA medium. The culture bottles were shaken gently daily to resuspend cells. Chlorophyll fluorescence was used as a proxy of biomass and measured directly by putting the culture bottles into the chamber of a fluorometer (Hitachi F-7000, Fluorescence spectrophotometer, Tokyo, Japan) at specified excitation and emission wavelengths of 525 and 680 nm respectively. The change in fluorescence values over time were then used to calculate the specific growth rates.

To compare physiological characteristics, strains were exposed to their growth-saturating light intensities in 250 mL conical flasks. After reaching the log phase, cells were inoculated in to each batch culture and the desired conditions were maintained. Sampling was performed between 9.00 a.m.–10.00 a.m. each time to avoid effects related to the light/dark cycle.

### 2.2. Cell Morphology and Trichome Length

Cell concentrations of *M. aeruginosa* were estimated by counting in a Neubauer Haemocytometer at 40× magnification on a Zeiss Axio Scope.A1, using samples fixed with Lugol’s iodine.

For filamentous strains, the number of trichomes mL^−1^ were determined from trichome length per mL and the mean number of cells per trichome according to Pierangelini et al. [23], using an improved Neubauer hemocytometer. Cell biovolume was calculated from at least 20 measurements on each of three independent biological replicates under each condition using the equation for a sphere: V = π/6 × d^3^, where d = diameter [24].

### 2.3. Pigment Analysis

For chl *a* determinations, cells were collected by centrifugation at 3400× *g* for 15 min, resuspended in 100% methanol and kept in the dark at 4 °C overnight. The suspension was centrifuged again and the supernatant was used to measure the absorbance at 632, 652, 654 and 696 nm using a Cary-50 UV-V spectrophotometer. Chl *a* concentration was then calculated using the equation of Ritchie [25]. Carotenoid concentrations were estimated using the same extract but with absorbance measured at 480 nm [26]. For estimation of phycobilins (phycocyanin (PC), allophycocyanin (APC) and phycoerythrin (PE)), cells were centrifuged at 3400× *g* for 15 min, resuspended in 0.05 M phosphate buffer (pH 6.7) then ruptured using a probe sonicator (BRANSON Model 102). The extract was centrifuged at 3400× *g* for 15 min and the supernatant used to measure the absorbance at 562, 615 and 652 nm. The equations of Bennett and Bogorad [27] were used to calculate PC, APC and PE concentrations.

### 2.4. Photosynthetic Parameters

Relative electron transport rate (rETR) as a function of irradiance, maximum quantum yield (F_v_/F_m_), light harvesting efficiency (α) and the light saturation parameter I_k_ were measured using a PHYTO-PAM phytoplankton analyser (Heinz Walz GmbH, Effeltrich, Germany). For all experiments relating to photosynthetic parameters measurements, cells were grown to exponential phase and kept in the dark for 15 min before a sample (3.5 mL) was taken and put in the chamber of PHYTO-PAM to carry out measurements of F_v_/F_m_. Rapid Light Curves (RLCs) were measured with 30 s intervals at each actinic light level. Maximum rates of relative electron transport (rETR_max_), α and I_k_ were calculated by using the instrument software for the cyanobacterial channel.

The PHYTO-PAM was also used to measure non-photochemical quenching (NPQ). Approximately 1 × 10^7^ cells were collected by centrifugation and resuspended in 3.5 mL fresh culture medium then dark acclimated for 15 min before being exposed to a saturating pulse to estimate maximum fluorescence [4] and the maximum quantum yield of photosynthetic energy conversion of PSII (F_v_/F_m_). The sample was then illuminated for 5–10 min with actinic light of 480 µmol photons m^−2^ s^−1^ and a 60 s cycle of saturating red pulses was applied until a stable value of maximum fluorescence yield in the light, F_m_′ was obtained. The Stern-Volmer equation was used to calculate NPQ as NPQ = (F_m_/F_m_′) − 1.

Light-saturated rates of photosynthetic oxygen evolution (P_max_) and dark respiration (R_d_) were measured using a Clark type O_2_ electrode (Hansatech, Norfolk, UK). Approximately 25 × 10^6^ cells were harvested from the culture, centrifuged (10 min, 3400× *g*) and the pellet resuspended in 2 mL of fresh HEPES-buffered (pH 6.3) medium containing 2 mM sodium bicarbonate. This suspension was then placed into an O_2_ electrode chamber for measurement of oxygen exchange rates. Prior to R_d_ and P_max_ measurements, the O_2_ concentration in the suspension was reduced to 20% of air saturation by bubbling with N_2_. R_d_ measurements were taken after dark incubation of the cell suspension for 5 min. Maximum O_2_ evolution rates of the culture were measured under a saturating light intensity previously determined from Phyto-PAM measurements.

### 2.5. Statistical Analysis

One way ANOVA followed by Tukey multiple comparison tests were performed to examine the statistical significance of variations among means between the strains. Comparison between two strains was tested by unpaired two tailed *t*-test with Welch’s correction of data. All the analyses were carried out using the statistical software GraphPad Prism 6, with a significance level at *p* < 0.05.

## 3. Results

### 3.1. Growth and Acclimation of Cyanobacteria at Different Light Levels

**Figure 1:**
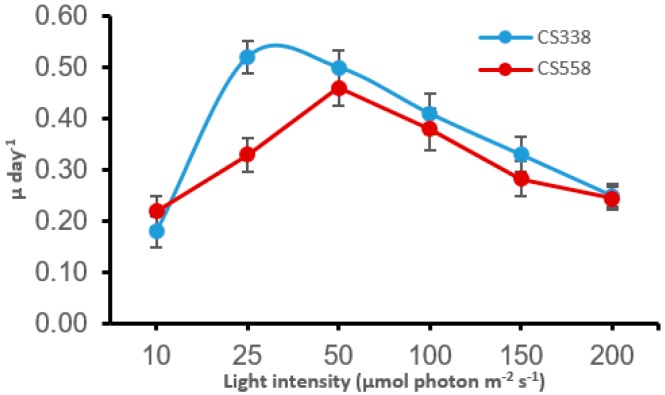
Effects of light intensity on specific growth rates (µ) of *Microcystis aeruginosa* strain CS338 and CS558. Red and blue symbols/lines are toxic and non-toxic strains respectively. Error bars represent the standard deviation of three replicates.

**Figure 2:**
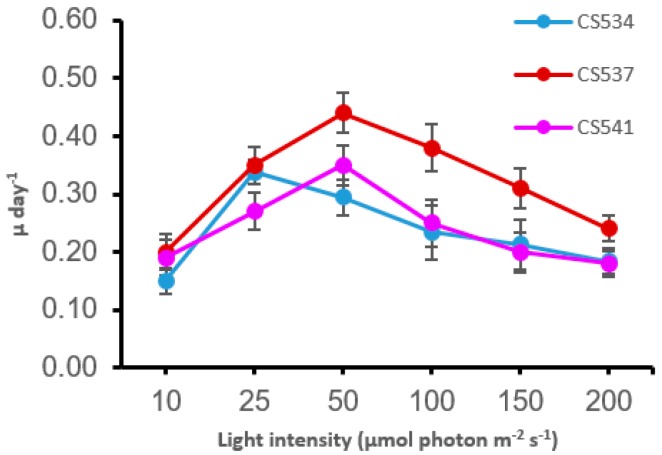
Effects of light intensity on specific growth rates (µ) of *Anabaena circinalis* strains CS534, CS537 and CS541. Red and purple symbols/line are toxic strains and blue is the non-toxic strain. Error bars represent the standard deviation of three replicates.

The growth rate and final cell concentrations of both species were strongly affected by light conditions. It is evident from Figure 1 and Figure 2 that the toxic strains of both species (CS558 for *M. aeruginosa* and CS537 and CS541 for *A. circinalis*) show saturation of growth (µ) at a higher light intensity compared to the non-toxic strains (CS338 for *M. aeruginosa* and CS534 for *A. circinalis*). For CS338, maximum µ was 0.55 day^−1^ at 25 µmol photon m^−2^ s^−1^ and for CS558 0.46 day^−1^ when grown at 50 µmol photon m^−2^ s^−1^. The maximum µ of strain CS537 (0.44 day^−1^) and CS541 (0.35 day^−1^) was observed at 50 µmol photon m^−2^ s^−1^ whereas strain CS534 (0.33 day^−1^) had a maximal growth rate at 25 µmol photon m^−2^ s^−1^.

### 3.2. Cell Biovolume

**Figure 3:**
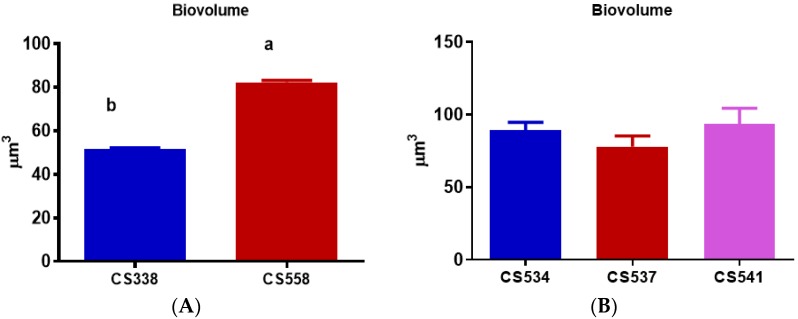
Cell biovolume of (**A**) *M. aeruginosa* strains CS338 and CS558 and (**B**) *A. circinalis* strains growing at their optimal light intensities (see text for details of these light levels). Samples were taken at exponential phase (day 5). Vertical bars indicate standard deviations of at least three replicates. Different letters above the bars indicate means that are significantly different at *p* < 0.05).

*M. aeruginosa* strains showed significant differences (*t*-test, *p* < 0.0001) in cell biovolume, with values almost 1.5 times higher in CS558 (toxic) compared to CS338 (non-toxic) strains (Figure 3A). On the other hand, strains of *A. circinalis* did not show any differences in biovolume (One-Way ANOVA: *F*_2,6_ = 2.806, *p* = 0.138) (Figure 3B).

Despite the difference in biovolume between toxic and non-toxic strains of *M. aeruginosa*, the pigment contents and physiological parameters described below showed the same trend when expressed per cell or per volume so only the per cell data are presented below.

### 3.3. Pigments

**Figure 4:**
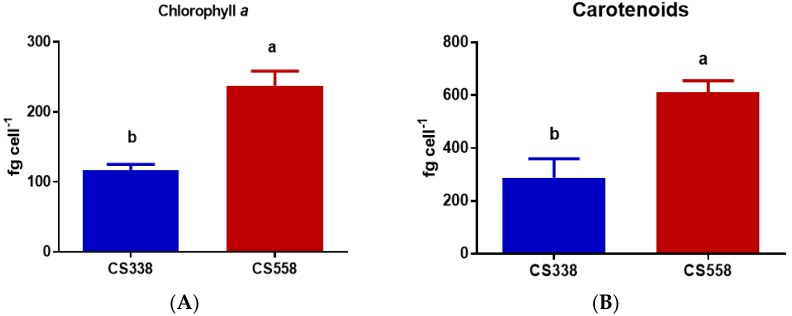

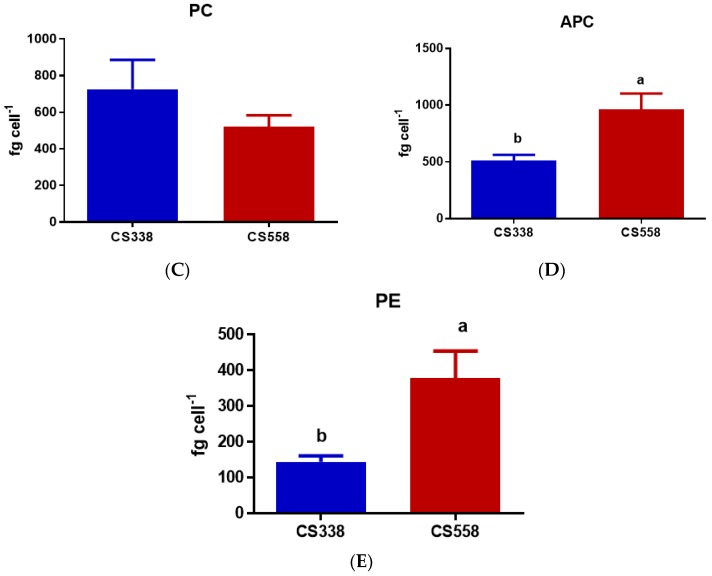
Cell concentrations of (**A**) chlorophyll *a*, (**B**) carotenoids, (**C**) phycocyanin, (**D**) allophycocyanin (APC), and (**E**) phycoerythrin (PE), in *M. aeruginosa* strains CS338 and CS558 growing in batch culture exposed at optimal light for growth. Samples were taken at exponential phase (day 5). Vertical bars indicate standard deviations of at least three replicates. Different letters above the bars indicate means that are significantly different at *p* < 0.05).

In *M. aeruginosa*, cellular concentrations of chlorophyll *a* (*t*-test, *p* = 0.0042) and carotenoids (*t*-test, *p* = 0.005) were higher in CS558 (toxic) compared to CS338 (non-toxic) (Figure 4A,B). Other strain-specific differences were observed for APC (*t*-test, *p* = 0.0198; Figure 4D) and PE (*t*-test, *p* = 0.0286; Figure 4E) where strain CS558 (toxic) contained higher values than strain CS338. No strain-specific differences were observed for PC (*t*-test, *p* = 0.1454; Figure 4C).

**Figure 5:**
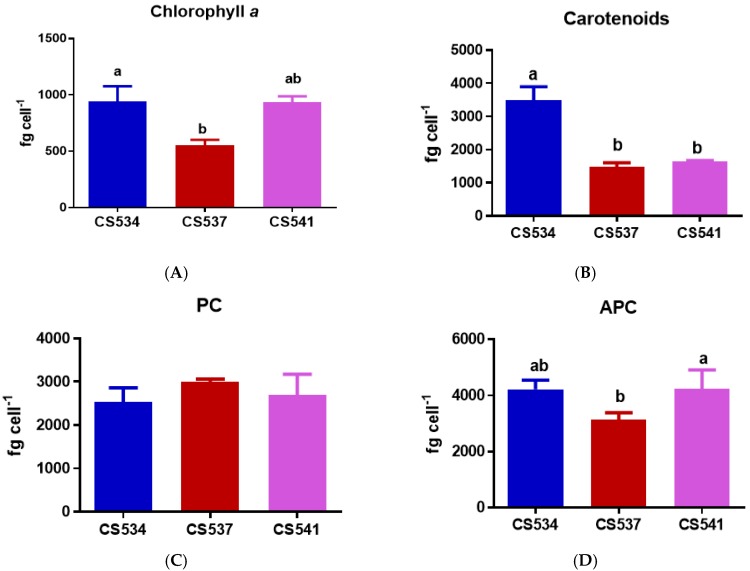

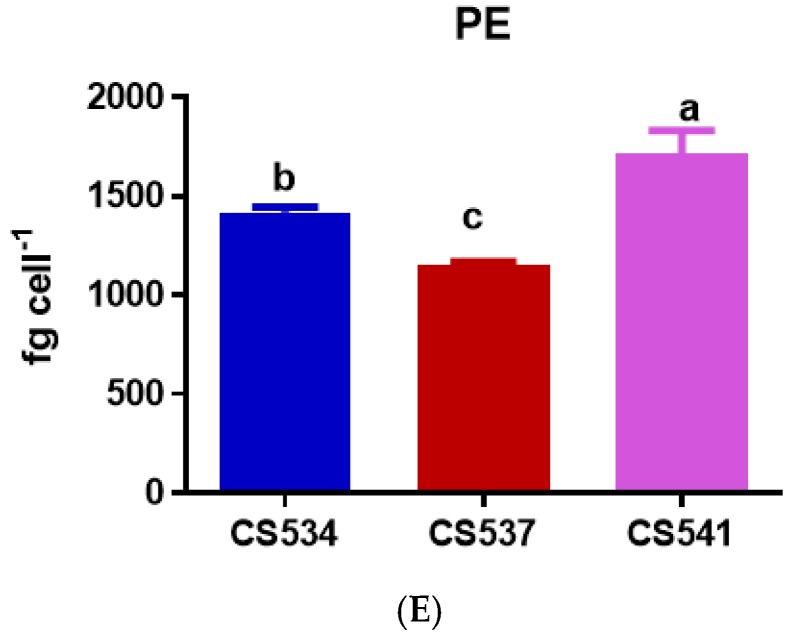
Cell concentrations of (**A**) chlorophyll *a*, (**B**) carotenoids, (**C**) phycocyanin, (**D**) allophycocyanin (APC), and (**E**) phycoerythrin (PE), in *A. circinalis* strains CS534, CS537 and CS541 growing in batch culture under optimal light for growth. Samples were taken at exponential phase (day 5). Vertical bars indicate standard deviations of at least three replicates. Different letters above the bars indicate means that are significantly different at *p* < 0.05).

In the case of *A. circinalis*, significant differences among the strains were observed in terms of cellular contents of chlorophyll *a* (One-Way ANOVA: *F*_2,6_ = 18.7, *p* = 0.0026, Figure 5A) and carotenoids (One-Way ANOVA: *F*_2,6_ = 62.17, *p* < 0.0001, Figure 5B). The chlorophyll *a* content was higher in CS534 (non-toxic) and CS541 (toxic) than in CS537 (toxic). The carotenoid content of the cells was significantly higher in strain CS534 (non-toxic) as compared to the other two strains. Other strain-specific differences were observed for APC (One-Way ANOVA: *F*_2,6_ = 5.601, *p* = 0.0424, Figure 5D) and PE (One-Way ANOVA: *F*_2,6_ = 46.13, *p* = 0.0002, Figure 5E) whereas PC showed no differences (One-Way ANOVA: *F*_2,6_ = 1.497, *p* = 0.297, Figure 5C) between the three strains.

### 3.4. Dark Respiration (Rd) and Photosynthesis

**Figure 6:**
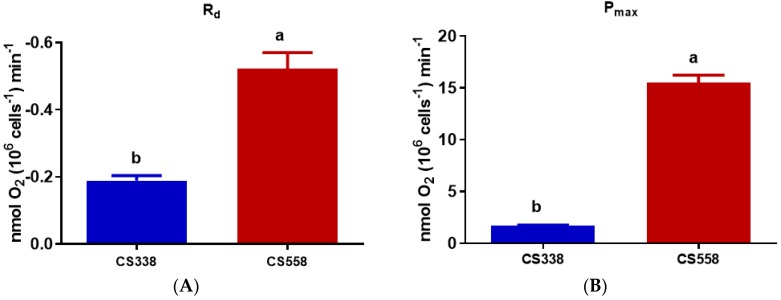

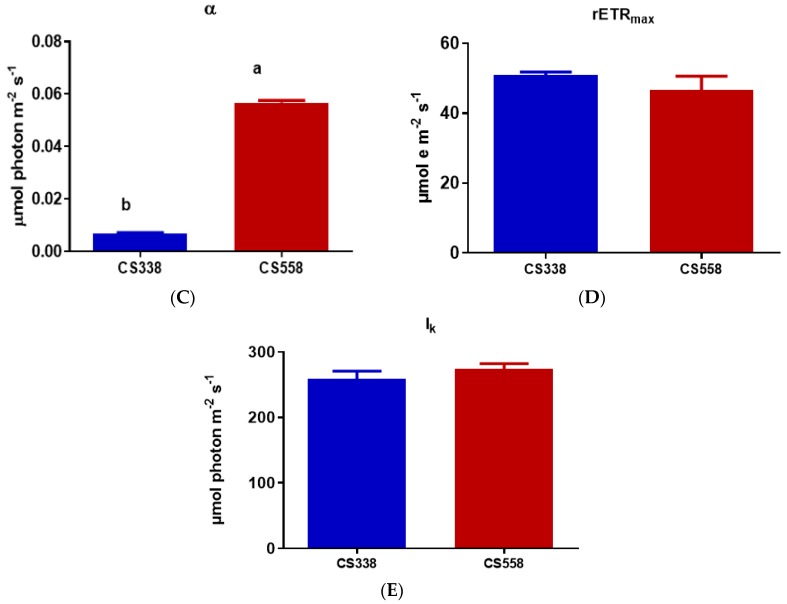
Comparison of dark respiration and photosynthesis in *M. aeruginosa* strains CS338 and CS558 growing in batch cultures at optimal light intensity. (**A**) dark respiration (R_d_); (**B**) maximum photosynthetic rates (P_max_); (**C**) light harvesting efficiency (α); (**D**) relative electron transport rate (rEtr_max_); (**E**) Light saturation parameter. Samples were taken at exponential phase (day 5). Error bars represent standard deviation of three replicates. Different letters above the bars indicate means that are significantly different at *p* < 0.05).

In case of *M. aeruginosa*, both R_d_ and P_max_ (maximum photosynthetic rate) showed significant differences (R_d_: *t*-test, *p* = 0.0037; P_max_: *t*-test, *p* = 0.0002) between strains (Figure 6A,B). Strain CS558 (toxic) showed lower R_d_ (0.522 nmol O_2_ 10^6^ cells^−1^ min^−1^) and higher P_max_ (15.530 nmol O_2_ 10^6^ cells^−1^ min^−1^) values than strain CS338 (R_d_, 1.887 nmol O_2_ 10^6^ cells^−1^ min^−1^, P_max_, 1.727 nmol O_2_ 10^6^ cells^−1^ min^−1^). These differences were not attributable to differences in biovolume as R_d_ and P_max_ expressed on a per µm^3^ basis showed the same trend (data not shown).

**Figure 7:**
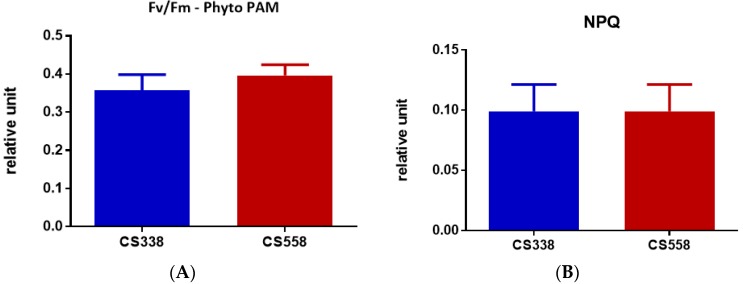
Comparison of both (**A**) maximum quantum yield (F_v_/F_m_) and (**B**) non-photochemical quenching (NPQ) of *M. aeruginosa* strains CS338 and CS558 growing in batch culture. Samples were taken at exponential phase (day 5). Error bars represent standard deviation of three replicates.

The light harvesting efficiency (α) showed significant differences between strains (*t*-test, *p* < 0.0001, Figure 6C). However, rETR_max_ (*t*-test, *p* = 0.1967, Figure 6D) and the light requirement for saturation of photosynthesis, I_k_ (*t*-test, *p* = 0.1524, Figure 6E) exhibited no significant differences between strains. Maximum quantum yield (F_v_/F_m_) (*t*-test, *p* = 0.2511, Figure 7A) and non-photochemical quenching (NPQ) also showed no variation across strains (*t*-test, *p* = 0.9999, Figure 7B).

**Figure 8:**
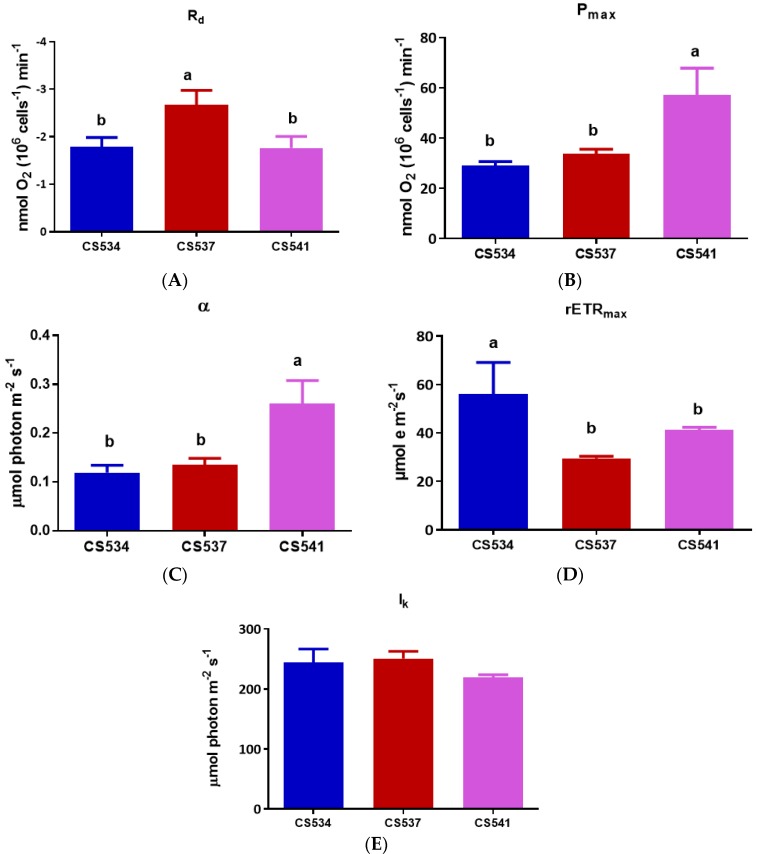
Comparison of both dark respiration and photosynthesis in *A. circinalis* strains CS534, CS537 and CS541 growing in batch culture exposed at the optimum light for growth. (**A**) dark respiration (R_d_); (**B**) maximum photosynthetic rates (P_max_); (**C**) light harvesting efficiency (α); (**D**) relative electron transport rate (rETR_max_); (**E**) light saturation parameter (I_k_). Samples were taken at exponential phase (day 5). Error bars represent standard deviation of three replicates. Different letters above the bars indicate means that are significantly different at *p* < 0.05).

For *A. circinalis*, differences in both R_d_ and P_max_ (Figure 8A,B) were observed between the three strains of this species. The R_d_ of CS537 (2.675 nmol O_2_ 10^6^ cells^−1^ min^−1^) was significantly higher than that of CS534 (1.791 nmol O_2_ 10^6^ cells^−1^ min^−1^) and CS541 (1.764 nmol O_2_ 10^6^ cells^−1^ min^−1^) (One-Way ANOVA: *F*_2,6_ = 13.03, *p* = 0.0066). The P_max_ values of the strains were also significantly different (One-Way ANOVA: *F*_2,6_ = 17.19, *p* = 0.0059). The value was almost double in CS541 (57.180 µmol photon m^−2^ s^−1^) compared to CS537 (33.86 nmol O_2_ 10^6^ cells^−1^ min^−1^) while in strain CS534 it was 28.97 nmol O_2_ 10^6^ cells^−1^ min^−1^.

**Figure 9:**
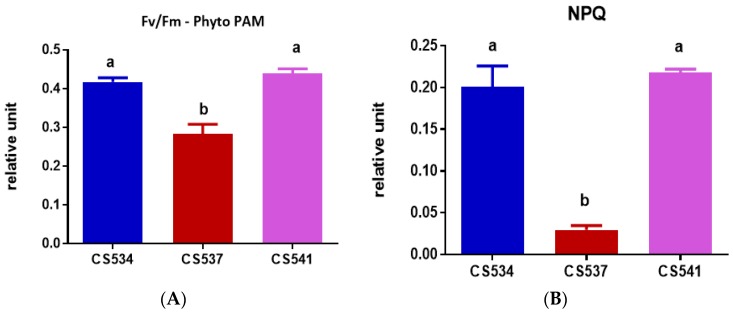
Comparison of both (**A**) maximum quantum yield (F_v_/F_m_) and (**B**) non-photochemical quenching (NPQ) of *A. circinalis* strains CS534, CS537 and CS541 growing in batch culture exposed at the optimum light intensity for growth. Samples were taken at exponential phase (day 5). Error bars represent standard deviation of three replicates. Different letters above the bars indicate means are significantly different at *p* < 0.05).

The light harvesting efficiency (α) (Figure 8C) was significantly higher (One-Way ANOVA: *F*_2,6_ = 20.05, *p* = 0.0022) in strain CS541 (0.2602) as compared to CS534 (0.1194) and CS537 (0.1354). However, the I_k_ (Figure 8E) values showed no significant differences (One-way ANOVA, *p* = 0.0952) between strains. The rETR_max_ (Figure 8D) also varied between strains (One-Way ANOVA: *F*_2,6_ = 9.122, *p* = 0.0152). Values for CS534 were significantly higher than for CS537 but not CS541. There was a significant difference (One-Way ANOVA: *F*_2,6_ = 71.87, *p* < 0.0001) in maximum quantum yield (F_v_/F_m_) (Figure 9A) between strains. The value for strain CS537 was lower compared to those of CS534 and CS541. Non-photochemical quenching (NPQ) also varied between strains with strain CS537 significantly lower than the other two strains (One-Way ANOVA: *F*_2,6_ = 150.5, *p* < 0.0001, Figure 9B).

## 4. Discussion

The results presented in this study clearly indicate differences in growth and physiological characteristics of toxic and non-toxic strains of *M. aeruginosa* and *A. circinalis* in relation to light climate. Strains of both species are able to survive very low light intensity (10 µmol photons m^−2^ s^−1^) to higher light intensity (200 µmol photons m^−2^ s^−1^). The ability of the strains to survive across a range from low to high light intensity reflects their dominance in Australian freshwater systems characterized by variable light intensity [23,28]. In terms of specific growth rate, the toxic strains of both species required higher light intensities to reach saturation compared to non-toxic strains. However, at low light the toxic strains grew faster than the non-toxic strains suggesting that the energetic cost of toxin production is not enough to influence growth rates under energy-limited conditions and the differences between strains may be related to factors other than toxin production. Although light is a significant factor controlling algal growth, its role in toxin production is mostly species specific. There is some evidence, however, that species’ toxin production varies between saturating, supra-saturating and limiting light intensities [13,14,15,16].

In the case of *M. aeruginosa*, cell biovolume was larger in strain CS558 (toxic) which might affect the light field. Big cells are more prone to self-shading with increasing cell concentration [29]. Due to their bigger cell size CS558 (toxic) contains higher Chl *a* and carotenoids compared to CS338 (non-toxic), though biovolume did not entirely account for this difference as similar trends were observed for chl *a* and carotenoids per unit cell volume. A similar result was found by Pierangelini et al. [23] for two strains of the cyanobacterium *Cylindrospermopsis raciborskii*. On the other hand, *A. circinalis* strains did not show any comparable differences based on cell biovolume. However, strain CS534 (non-toxic) contained relatively high chl *a* and carotenoid concentrations in cells relative to other strains. Among the other parameters APC and PE showed significant differences with the strain CS537 containing relatively low values than the other two strains.

The parameter α represents the light harvesting efficiency and the photosynthetic energy conversion efficiency [30] of cells. In some cases there are correlations found between the value of α and cellular pigment composition/content under different light intensities [31,32]. The lower α value of CS338 (non-toxic) as compared to CS558 (toxic) could be due to a lower Chl *a* to biovolume ratio, which suggests a poor ability to harvest light in low light conditions. Similar results were also found for *A. circinalis* strain CS537 (toxic). 

In the case of *M. aeruginosa*, the higher values of both R_d_ and P_max_ in strain CS558 (toxic) could be partly due to the higher cell biovolume compared to CS338 (non-toxic) which results in higher respiratory consumption and photosynthetic production of oxygen, but similar trends between strains were observed in respiratory and photosynthetic rates per unit volume. However, the higher P_max_ value of CS541 (toxic) of *A. circinalis* cannot be explained in the same terms as there were not any comparable differences in cell biovolume between the strains. 

F_v_/F_m_ is a measure of the physiological status of PSII in algae/plants. The F_v_/F_m_ values observed for strains of both species are within the range for unstressed cyanobacteria [33]. No differences were observed between strains in the case of *M. aeruginosa,* but *A. circinalis* showed significant differences between strains, with strain CS537 (toxic) having a lower F_v_/F_m_ compared to the other two strains (one toxic, one non-toxic). 

In cyanobacteria, NPQ is associated with dissipation of excess light energy as heat, i.e., it operates as a photoprotective mechanism [34,35] and can also reflect state transitions i.e., regulation of excitation energy transfer between phycobilisome/PSII and PSI [36,37]. The lower value of F_v_/F_m_ in the case of *A. circinalis* strain CS537 (toxic) as compared to the other strains could be explained by the fact that this particular strain might be slightly stressed, but it is hard to conclude this with any certainty. If toxin production were high enough to act as a significant electron sink, using ATP/NADPH at a higher rate and thus recycling ADP/NADP^+^ more rapidly, we would expect less stress on PSII and higher values of F_v_/F_m_ and less need to up-regulate NPQ. However, toxicity of the strains did not appear to be related to the differences in F_v_/F_m_ and NPQ. Thus, we propose that the NPQ measured in *M. aeruginosa* and *A. circinalis* is more likely to be driven by state transitions [37] rather than by heat dissipation, as described by Pierangelini et al. [23].

In conclusion, this study reveals differences in growth and physiology of strains within species and between species. The results suggest that different strains respond differently at different light intensities. Toxic strains tend to have required higher light for growth. Although some differences in physiological characteristics were observed between species and strains there were not any consistent trends in relation to toxicity, and we acknowledge that factors other than toxin production may also be involved in the observed strain differences. Changes in light availability may affect bloom intensity of toxic and non-toxic strains of cyanobacteria and also the amount of toxin produced. Further studies with a greater range of strains of differing toxicity would be helpful in elucidating this issue.
